# Supplementary material for: Whole-brain meso-vein imaging in living humans using fast 7-T MRI
Source: Sci Adv. 2026 Jan 9;12(2):eaea4540. doi: 10.1126/sciadv.aea4540 (PMC12787541; doi:10.1126/sciadv.aea4540)
Supplement: Supplementary file 1 — Figs. S1 to S5 Legends for movies S1 and S2 [file sciadv.aea4540_sm.pdf]

Science Advances
AAAS

Supplementary Materials for

Whole-brain meso-vein imaging in living humans using fast 7-T MRI

Omer Faruk Gulban *et al.*

Corresponding author: Omer Faruk Gulban, farukgulban@gmail.com; Dimo Ivanov, dimozfn@gmail.com

Sci. Adv. **12**, eaea4540 (2026)
DOI: 10.1126/sciadv.aea4540

The PDF file includes:

Figs. S1 to S5
Legends for movies S1 and S2

Other Supplementary Material for this manuscript includes the following:

Movies S1 and S2

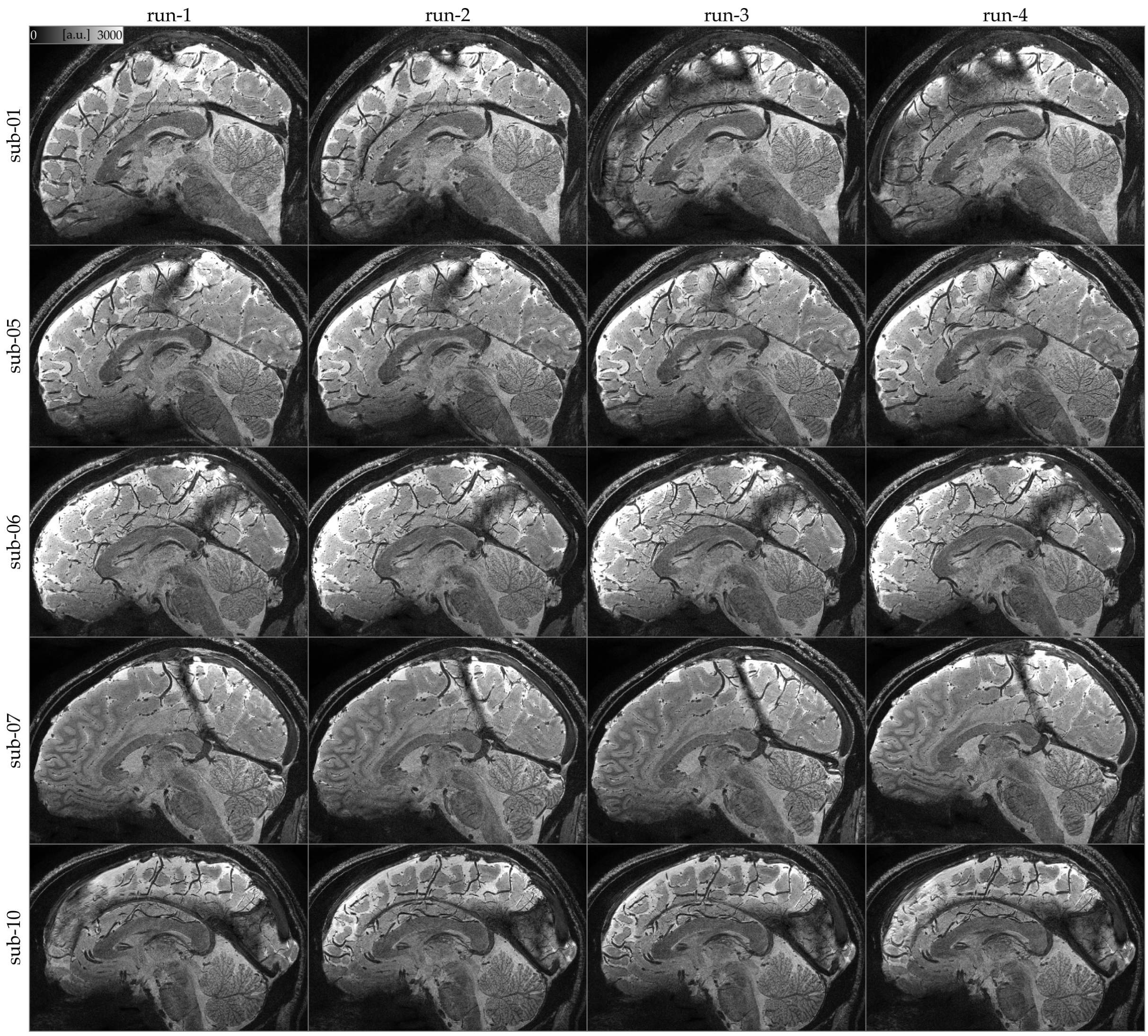

Supplementary Figure 1: Imaging slab positioning and data quality. No data were discarded due to bulk head motion within acquisitions (see (*16*), Supplementary Figure 3 for an example of within-run head motion). Notably, a substantial between-run head motion of nearly a centimeter (measured from the inferior frontal region) was observed in sub-07 run-04. However, this did not noticeably degrade image quality, particularly in resolving fine details of mesoscopic veins. While anecdotal, this finding highlights the effectiveness of our participant preselection strategy and the importance of explicit between-run instructions, such as encouraging participants to move if uncomfortable. Allowing controlled movement in between acquisitions increases the likelihood of obtaining high-quality images. Note that our images are acquired consecutively within a short period of time within 2024 and the gaps in subject numbers do not reflect any data removal.

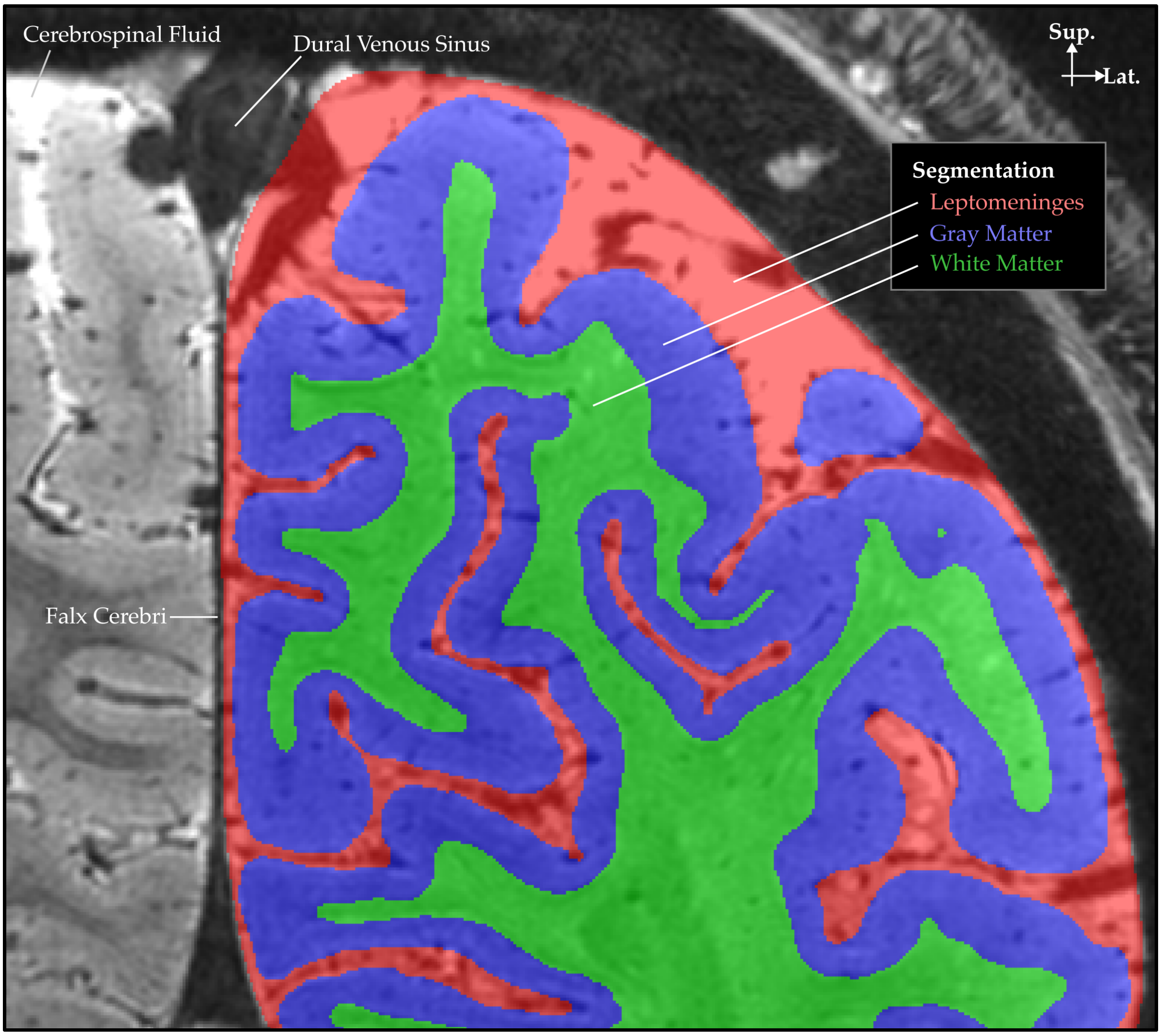

Supplementary Figure 2: Segmented tissues overlaid on T_2^*-weighted images for sub-01's left hemisphere. In our T_2^*-weighted images cerebrospinal fluid appears very bright, veins and large arteries appear very dark, cortical gray matter appears brighter than white matter. Manual edits on the initial segmentation were done using ITKSNAP v4.2.2 to improve the accuracy and precision of each tissue label. A conjunction mask of leptomeninges, gray matter, and white matter was applied to exclude other tissues before voxel value rendering in BrainVoyager v24.0 (see **Figure 5-7**). Gray matter segmentation was used to compute geometric layers via the LN2_LAYERS program in LayNii v2.7.0, which were subsequently used to reconstruct triangular meshes in BrainVoyager (see **Figures 8-9**).

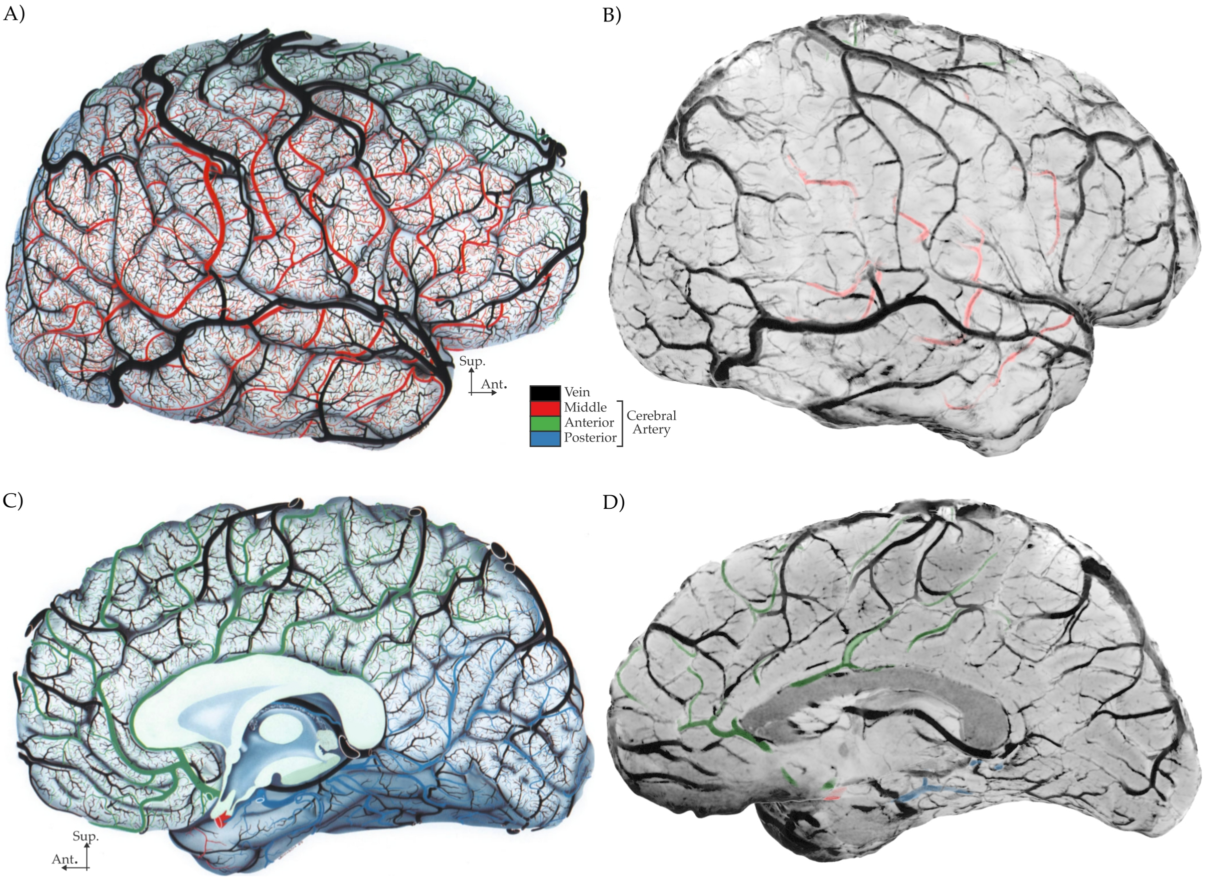

Supplementary Figure 3: Comparison of cortical angioarchitecture visualized using post-mortem ink injection (Panels A and C, adapted from (*31*)) and in vivo T_2^*-weighted MRI (Panels B and D). Note that smaller veins require a more zoomed in view to be visible on our in vivo images (see Figures 8-10). To be more comparable to Jean-Louis Vannson's drawings, we inverted the intensity channel in our $1/T_2^*$-weighted volume-rendered images, rendering veins dark and cortical gray matter bright, while color-coding large arteries. Panels A and B are adapted from Figures 233 and 234 in (*27*) (used with permission of "Springer Nature BV", from "The human brain: surface, three-dimensional sectional anatomy with MRI, and blood supply, Duvernoy, Henri M., Bourgouin, P., Second completely revised and enlarged edition, 1999"; permission conveyed through Copyright Clearance Center, Inc.).

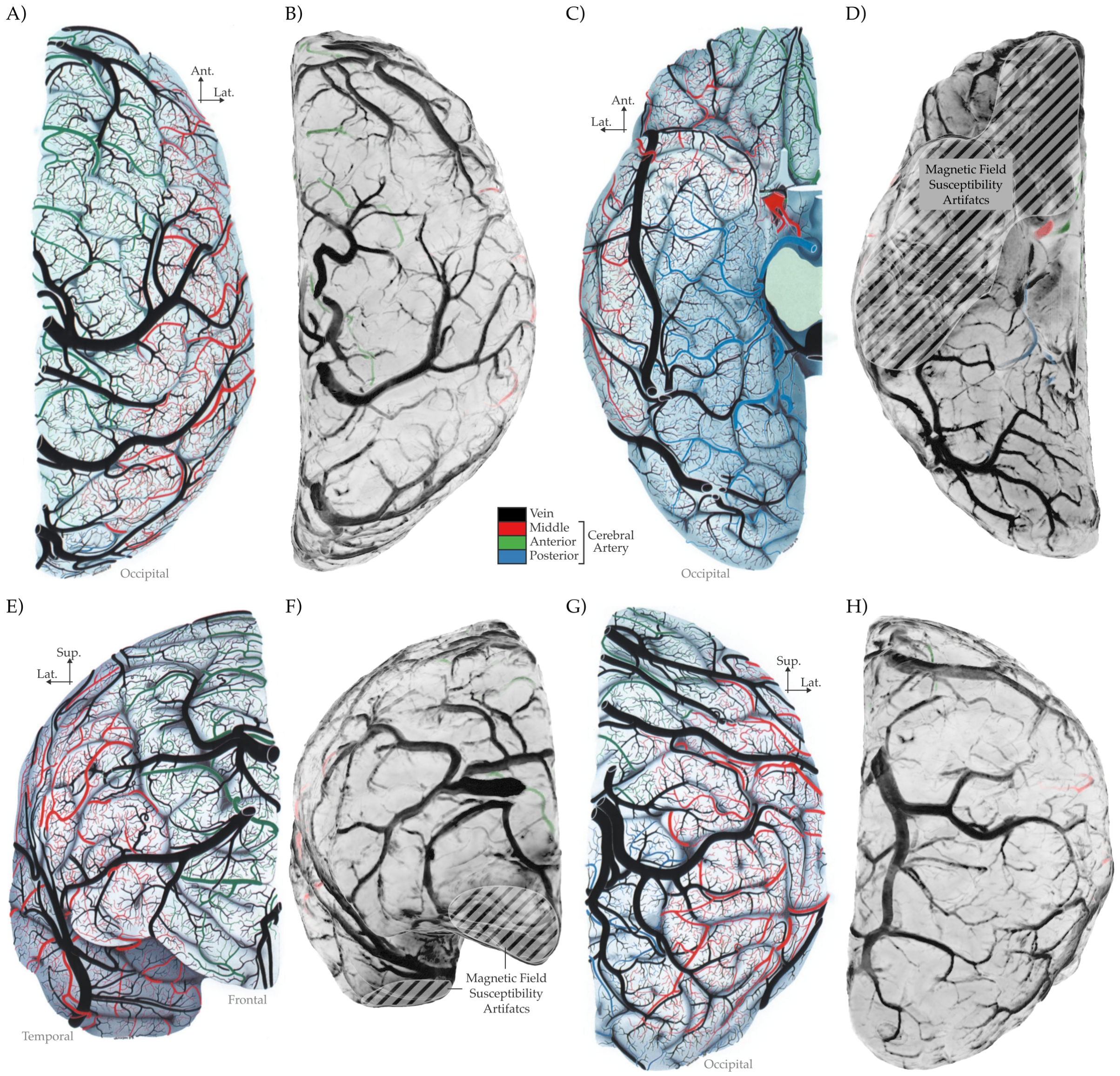

Supplementary Figure 4: Comparison of comparing cortical angioarchitecture visualized using postmortem ink injection (Panels A, C, E, G, adapted from (27)) and in vivo T_2^*-weighted MRI (Panels B, D, F, H). Format same as Figure 5. In addition, we have marked the inferior brain regions affected by magnetic field susceptibility artifacts, where tissue segmentation becomes unreliable. Panels A, C, E, G are adapted from Figures 235-238 in (27) (used with permission of "Springer Nature BV", from "The human brain: surface, three-dimensional sectional anatomy with MRI, and blood supply, Duvernoy, Henri M., Bourgouin, P., Second completely revised and enlarged edition, 1999"; permission conveyed through Copyright Clearance Center, Inc.).

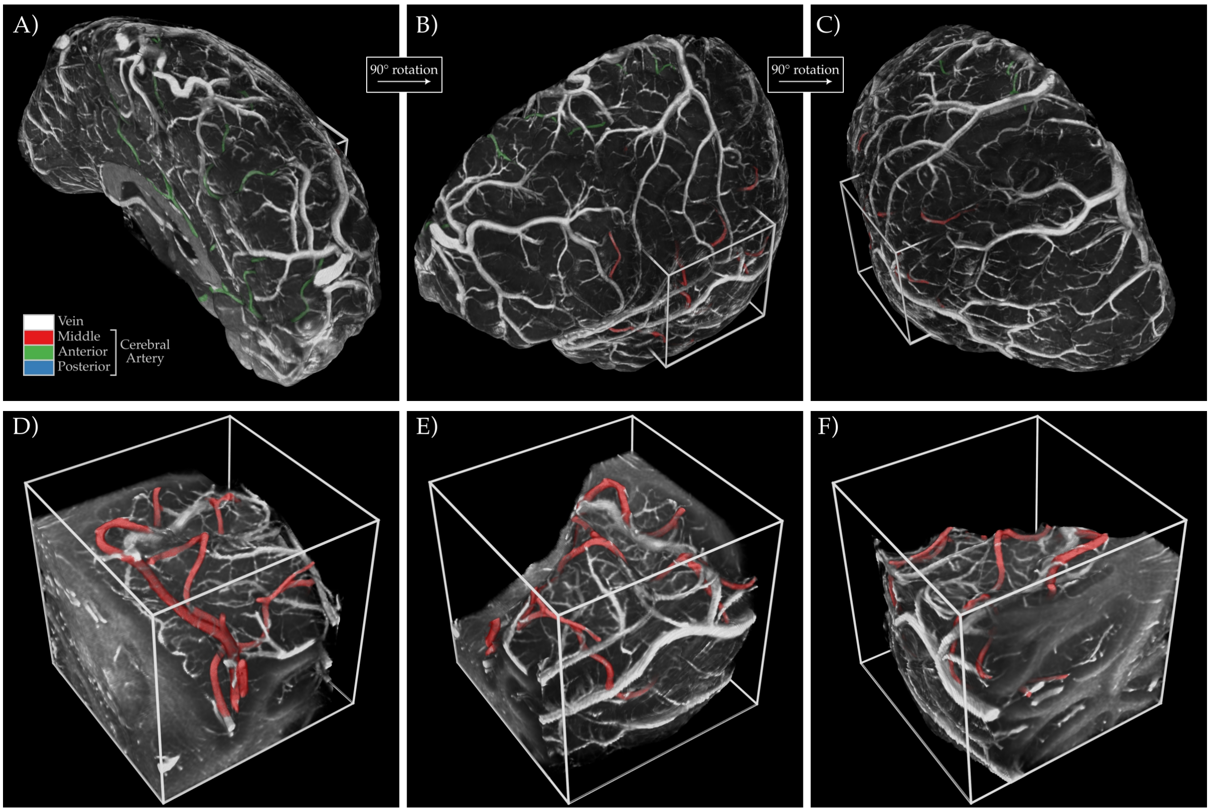

Supplementary Figure 5: Different perspectives of the whole brain (A-B), and zoomed-in, cutouts (D-F) for temporal lobe adjacent vessels of Figure 7. Bright tubular structures are leptomeningeal and pial veins. The colored cerebral arteries are classified by tracing their branches towards the brainstem.

Supplementary Video 1. The first animation shows volume rendering of leptomeningeal vessels of a human brain hemisphere. The second animation shows mid-thickness surface mesh. Both animations visualize $1/T_2^*$-weighted contrast.

Supplementary Video 2.This animation shows inflation of the mid-thickness cortical surface showing T_2^*-weighted contrast. Note that the intracortical meso-veins are visible as dark dots spread across the surface, both within gyri and sulci.
